# US28 Is a Potent Activator of Phospholipase C during HCMV Infection of Clinically Relevant Target Cells

**DOI:** 10.1371/journal.pone.0050524

**Published:** 2012-11-29

**Authors:** William E. Miller, William A. Zagorski, Joanna D. Brenneman, Diana Avery, Jeanette L. C. Miller, Christine M. O’Connor

**Affiliations:** 1 Department of Molecular Genetics, Biochemistry, and Microbiology, University of Cincinnati College of Medicine, Cincinnati, Ohio, United States of America; 2 Section of Virology, Department of Molecular Genetics, Lerner Research Institute, The Cleveland Clinic, Cleveland, Ohio, United States of America; University of Florida, United States of America

## Abstract

Members of the cytomegalovirus family each encode two or more genes with significant homology to G-protein coupled receptors (GPCRs). In rodent models of pathogenesis, these viral encoded GPCRs play functionally significant roles, as their deletion results in crippled viruses that cannot traffic properly and/or replicate in virally important target cells. Of the four HCMV encoded GPCRs, US28 has garnered the most attention due to the fact that it exhibits both agonist-independent and agonist-dependent signaling activity and has been demonstrated to promote cellular migration and proliferation. Thus, it appears that the CMV GPCRs play important roles in viral replication *in vivo* as well as promote the development of virus-associated pathology. In the current study we have utilized a series of HCMV/US28 recombinants to investigate the expression profile and signaling activities of US28 in a number of cell types relevant to HCMV infection including smooth muscle cells, endothelial cells and cells derived from glioblastoma multiforme (GBM) tumors. The results indicate that US28 is expressed and exhibits constitutive agonist-independent signaling activity through PLC-β in all cell types tested. Moreover, while CCL5/RANTES and CX3CL1/Fractalkine both promote US28-dependent Ca^++^ release in smooth muscle cells, this agonist-dependent effect appears to be cell-specific as we fail to detect US28 driven Ca^++^ release in the GBM cells. We have also investigated the effects of US28 on signaling via endogenous GPCRs including those in the LPA receptor family. Our data indicate that US28 can enhance signaling via endogenous LPA receptors. Taken together, our results indicate that US28 induces a variety of signaling events in all cell types tested suggesting that US28 signaling likely plays a significant role during HCMV infection and dissemination *in vivo*.

## Introduction

Cytomegaloviruses are members of the β -herpesviridae family and are characterized by their ability to replicate in a wide range of cell types and subsequently establish a life-long persistent infection in their hosts [1], [2]. The prototypical member of this family, human cytomegalovirus (HCMV), is present in a latent or persistent form in ∼50–75% of the human population [3]. Most healthy individuals harboring HCMV are asymptomatic, however the virus is the leading infectious cause of birth defects, causes significant morbidity in immunocompromised patients, and has been implicated as a co-factor in the progression of cardiovascular disease and malignancies such as glioblastoma multiform (GBM) [4], [5], [6], [7]. The gene products required for viral replication and promotion of disease *in vivo* as well as their mechanism(s) of action remain largely unknown.

The cytomegaloviruses each contain two or more genes that are homologous to the chemokine family of G-protein coupled receptors (GPCRs) [8], [9], [10], [11], [12], [13]. For example, HCMV encodes four GPCR homologues including US27, US28, UL33 and UL78, while murine cytomegalovirus (MCMV) encodes two GPCR homologues termed M33 and M78 [9], [11], [13], [14]. The most studied of the CMV GPCRs are HCMV US28 and its functional orthologue MCMV M33 [15], [16], [17], [18], [19], [20]. Although these genes are not required for viral replication in cell culture, both of these genes appear to play important roles during viral infection *in vivo* [20], [21], [22]. Using a combination of viral mutants and overexpression studies, HCMV-US28 has been reported to direct chemotaxis of vascular smooth muscle cells and macrophages in Boyden chamber migration assays suggesting that US28 may facilitate viral dissemination *in vivo* and/or exacerbate HCMV associated vascular disease [20], [23], [24]. In transgenic mice and in overexpression studies, US28 enhances cellular proliferation and is pro-tumorigenic, potentially implicating this gene in CMV-associated malignancies such as GBM [19], [25], [26], [27]. MCMV-M33 and its signaling activity is involved in viral replication in sites of persistence *in vivo* as MCMV recombinants deleted for M33 fail to grow in the salivary glands of mice [28], [29]. Taken together, these studies suggest that CMV GPCRs like M33 and US28 play important roles in pathogenesis *in vivo* by promoting viral dissemination and/or tissue specific viral replication and that a by-product of this activity may be the development of virus associated pathologies such as the acceleration of atherosclerosis or potentiation of cancer.

US28 is highly related to the β -chemokine receptors CCR1 and CCR5 and accordingly binds with nanomolar affinity to several chemokines including CCL5 (RANTES), CCL2 (MCP-1), and CX3CL1 (Fractalkine) [13], [14], [30], [31], [32], [33]. Although US28 binds to these chemokines, it exhibits high levels of agonist-independent signaling activity [15], [34], [35]. This agonist-independent signaling activity results in the constitutive activation of Gαq, phospholipase C- β (PLC-β) and a number of transcription factors [15], [18], [35], [36], [37]. Moreover, experiments in mouse embryonic fibroblasts (MEFs) deficient in Gα_q/11_ have provided solid genetic evidence demonstrating the role of Gα_q/11_ in mediating US28 driven PLC-β activity [38]. In contrast, US28 mediated release of intracellular calcium, activation of the small G-protein Rho, and activation of the Focal Adhesion Kinase (FAK) only occurs following chemokine binding, suggesting that the interaction of ligands with US28 does in fact induce a conformational change in the receptor that activates a subset of the US28-driven signaling pathways [14], [17], [23], [24]. A combination of both agonist-independent and agonist-dependent signaling activity appears to be important for the biologic effects of US28 including smooth muscle cell migration [15], [18], [20].

Additionally, the CC and CX3C chemokines appear to have differential effects on US28. For example, CCL5/RANTES has been shown to behave as both a neutral agonist, having no measurable effect on constitutive PLC-β signaling or transcription factor activation, and as a positive agonist leading to the release of intracellular calcium and activation of FAK [14], [17], [35]. CX3CL1/Fractalkine has been suggested to behave as an inverse agonist, as it significantly blocks the ability of US28 to promote PLC-β activity, and as a positive agonist leading to the release of intracellular calcium and activation of FAK [15], [18], [30], [32], [33]. Moreover, CCL5/RANTES and CX3CL1/Fractalkine have been reported to exhibit cell-specific effects as CCL5/RANTES promotes smooth muscle cell migration and inhibits macrophage migration while CX3CL1/Fractalkine promotes macrophage migration and inhibits smooth muscle cell migration [39], [40]. Taken together, these data indicate that the mechanisms involved in the regulation of agonist-dependent and agonist-independent signaling as well as the influence of the cellular background on US28-dependent signaling remain poorly understood.

While US28 is the best studied of the HCMV GPCRs to date, this work has largely utilized ectopic and transgenic expression systems to evaluate US28 signaling. Only a handful of studies have used viral mutants to investigate US28 signaling in the context of acute HCMV infection and although informative, these studies were largely restricted to the human fibroblast model system. While the human fibroblast model is a powerful system to investigate many aspects of HCMV biology, it is clear that HCMV infects and replicates in a number of other cell types including cells of smooth muscle, endothelial, epithelial, astroglial/neuronal, and myeloid origin. In the current study we report our findings indicating that US28 is highly expressed during acute infection of a number of clinically relevant cell types and that it exhibits a unique pattern of agonist-independent and agonist-dependent signaling activities in these cells. These studies provide essential data regarding US28 activity in a variety of HCMV target cells and offer important insights into how this potent HCMV signaling molecule may ultimately impact HCMV pathogenesis *in vivo*.

## Materials and Methods

### Cell Culture and Virus Propagation

Human foreskin fibroblasts (HFFs) and MRC5 fibroblasts were purchased from ATCC. U373MG glioblastoma cells [41] were the gift of Timothy Kowalik at the University of Massachusetts. These cells were maintained in Dulbecco’s modified Eagle’s medium (Hyclone) supplemented with 10% Fetal Clone III serum (Hyclone) and 1% penicillin/streptomycin (Hyclone). Human Coronary Artery Smooth Muscle cells (HASMCs) and human umbilical vein endothelial cells (HUVECs) were obtained from ScienCell Research Laboratories (Carlsbad, Ca) and maintained in the manufacturer’s supplied medium. Cells were grown at 37°C in a humidified atmosphere with 5% CO_2_, split 1∶2 twice weekly, and used between passages 3 and 20. For propagation of HCMV, HFFs were infected at an MOI of 0.01 and fed every three days until the CPE reached 100%. Cell culture supernatant containing virus was harvested, snap frozen, and stored at −80°C until use.

### Construction of the US28 Viral Mutants and Reconstitution of Infectious Viruses

Bacterial artificial chromosomes (BAC) containing either the wild-type VR1814 or TB40/E strains of HCMV (FIX-BAC or TB40/E-BAC, clone #4) were obtained from Gabriella Hahn or Christian Szinger [42], [43]. FIX-based viruses in which the US28 gene was deleted or replaced by Flag-tagged WT, ΔN, or R129A mutants has been previously described [38], [43]. TB40/E-BAC was previously engineered to express mCherry as a marker for infection, termed TB40/E*wt*-mCherry [44]. This backbone was then used to generate a recombinant virus (TB40/E-US28Flag) that contained three tandem FLAG epitopes at the C-terminus of US28, as previously described [44]. In brief, the FLAG epitopes and Kan-frt cassette were PCR-amplified from pGTE-3xFLAG-Kan-frt [44] using primers listed in Table S1. The resulting product was then used to substitute the epitope tag for the Kan-frt cassette utilizing linear recombination [44], [45]. TB40/E-US28Flag was then used to generate two, independent US28 deletion mutants using *galK* recombineering techniques, described previously [46]. In brief, *galK* was PCR-amplified using primers listed in Table S1. The resulting PCR product was used to transform recombination-competent SW105 *E.* coli containing TB40/E-US28Flag. *GalK*-positive clones were selected and electroporated with complementary, annealed double-stranded (ds) oligos (Table S1). Mutants were then counterselected against *galK* and two mutants (TB40/E-ΔUS28-1 and TB40/E-ΔUS28-2) were validated for sequencing. TB40/E*wt*-mCherry [44] was used to generate two independent, recombinant viruses lacking all four ORFs encoding the GPCRs, termed TB40/E-ΔALL-1 and TB40/E-ΔALL-2, using *galK* recombineering as above. In brief, the ORFs encoding UL33 and UL78 were sequentially deleted by *galK* recombineering and validated by sequencing, as described above. Finally, the ORFs encoding US27 and US28 were deleted in tandem by *galK* recombineering techniques and validated by sequencing, as above. The primers and complimentary ds oligos used to generate TB40/E-ΔALL-1 and TB40/E-ΔALL-2 are listed in Table S1.

For reconstitution of recombinant viruses, 2×10^5^ MRC-5 cells were plated in six-well plates and transfected with 2 µg of FIX-BAC DNAs using either Superfect (Qiagen) or Transit IT (Mirus) lipid transfection reagents according to the manufacturer’s protocol. Ten days post- transfection, MRC-5 cells were transferred to T-25 flasks and fed with fresh media every 3–4 days. After the appearance of virus associated cytopathic effects (CPE), infected MRC-5 cells were mixed with uninfected HFFs and cultured until the CPE reached 100%. Supernatants containing recombinant viruses were used for the generation of virus stocks.

### Inositol Phosphate Accumulation

Cells were seeded in 12-well plates at a density of 1.5×10^5^ cells per well and either mock-infected or infected with HCMV recombinants at increasing MOIs. Twenty-four hours post-infection (hpi), media containing virus was removed and replaced with serum-free Modified Eagles Medium (MEM; Mediatech) containing 2 µCi/ml of [^3^H]myo-inositol (PerkinElmer Life Sciences). Forty-eight hpi, media was removed and replaced with serum free media supplemented with 20 mM LiCl for 3 hours. For samples stimulated with 10 nM CCL5 (Peprotech) or 10 nM CX3CL1 (Peprotech), the ligand was added to the appropriate concentration at the time the media was replaced with serum free media supplemented with LiCl. Reactions were stopped by aspirating medium, adding 1 ml of 0.4 M perchloric acid, and cooling undisturbed at 4°C for 5 min. 800 µl of supernatant was neutralized with 400 µl of 0.72 M KOH/0.6 M KHCO_3_ and subjected to centrifugation. 1 ml of supernatant was diluted with 3 ml of distilled H_2_O and applied to freshly prepared Dowex columns (AG1-X8; Bio-Rad). Columns were washed two times with distilled H_2_O; total inositol phosphates were eluted with 4.0 ml of 0.1 M formic acid, 1 M ammonium formate; and eluates containing accumulated inositol phosphates were counted in a liquid scintillation counter. 50 µL of neutralized supernatant was counted in a liquid scintillation counter to measure total incorporated [^3^H]myo-inositol. Data is expressed as accumulated inositol phosphate over total incorporated [^3^H]myo-inositol.

### Immunoprecipitation and Western Blotting

Cells were seeded in 100-mm dishes at a density of 2.0×10^6^ cells per plate and either mock-infected or infected with HCMV recombinants at a MOI of 3. Forty-eight hpi, cells were lysed in RIPA Buffer (150 mM NaCl, 10 mM Tris, 5 mM EDTA, 0.1% SDS, 1.0% DOC, 1.0% Triton X-100 and Complete™ protease inhibitors [Roche]). A small sample of the clarified lysate was saved as whole cell extracts and the remainder was incubated with anti-FLAG M2-agarose beads (Sigma) to immunoprecipitate FLAG-US28 proteins. Beads were washed twice with lysis buffer and eluted using 50 µL of Laemmli sample buffer. Whole cell extracts or FLAG immunoprecipitates were separated by SDS-PAGE and subjected to western blotting using antibodies directed against the FLAG epitope (sc-805; Santa Cruz); HCMV IE proteins (MAB810, Chemicon) or HCMV pUL44 (a kind gift from John D. Shanley; University of Connecticut). Reactive proteins were detected using the appropriate secondary antibodies in an enhanced chemiluminescence system (ECL; Amersham Biosciences).

### Measurement of Intracellular Calcium

Cells (HFFs, HASMC, U373MG) were infected with HCMV recombinants at an MOI of 3 for 48 hours, then loaded with Fluo4-AM (Molecular Probes) for 60 min at 37°C. Pertussis toxin (PTx) was used at a concentration of 200 ng/mL to treat infected cells 24 hours prior to loading (List Biological Laboratories). After basal levels of fluorescence were attained for 20 seconds, cells were stimulated 10 nM CCL5/RANTES or 10 nM CX3CL1 Fractalkine (Peprotech), and fluorescence was measured using an excitation wavelength of 488 nm and an emission wavelength of 505–515 nm every 3 seconds for 180–300 seconds using a FlexStation II Analyzer (Molecular Devices). Data is representative of at least three experiments performed in duplicate.

### Viral Growth Analysis

Multi-step growth analysis was performed on HFFs (MOI of 0.01 TCID_50_/cell) and HUVECs (MOI of 0.1 TCID_50_/cell) by infecting 2.5×10^5^ cells per well with either TB40/E*wt*-mCherry, TB40/E-ΔUS28, or TB40/E-ΔALL. Media was collected at various times post-infection and stored at −80°C. HFFs were used to titer infectious virus by evaluating the production of IE1 positive cells 24 hpi, using an antibody directed at IE1 (clone 1B12) [47] and a secondary antibody conjugated to Alexa-488 (Molecular Probes). As described previously, three random fields were quantified [44], [48]. Each experiment was performed in triplicate.

## Results

### pUS28 is Expressed in a Variety of Cell Types Important for *in vivo* Infection

To investigate US28 expression and signaling during acute infection of clinically important HCMV target cell types, we first constructed a number of clinical-isolate based HCMV derivatives to facilitate these studies and allow for infection of cells with restricted tropism such as endothelial cells. The HCMV derivatives and a description of the genetic modifications contained within each virus are listed in Table 1. The FIX-based derivatives were characterized in primary human fibroblasts (HFFs) in earlier publications from our laboratory [38], [43], while the TB40/E-based viruses were recently constructed and are reported for the first time in the current study. Briefly, the FIX-based derivatives include an otherwise wild-type virus containing a Flag-tagged US28 gene (FIX-US28Flag), a virus that eliminates the entire US28 gene (FIX-ΔUS28), a virus that deletes the US28 chemokine binding domain (FIX-US28ΔNFlag), and a virus that cripples US28 coupling to G-proteins (FIX-US28R129AFlag) [38], [43]. The TB40/E-based viruses include derivatives that contain a Flag-tagged US28 gene (TB40E-US28Flag), eliminate the entire US28 gene (TB40/E-ΔUS28), or delete all four of the HCMV-encoded GPCRs (TB40/E-ΔALL).

**Table 1:** Mutant viruses used in this study.

Virus	Genetic Modification in Virus
FIX-US28Flag	US28 gene contains Flag tag at amino terminus
FIX-ΔUS28	US28 gene deletion
FIX-US28ΔNFlag	US28 gene contains Flag tag at amino terminus and is deleted for the chemokine binding domain between amino acids 2 and 22
FIX-US28R129AFlag	US28 gene contains Flag tag at amino terminus and contains a mutation in the G-protein coupling motif that converts amino acid 129 from Arg to Ala
TB40/E*wt*-mCherry	BAC derived, contains SV40 driven mCherry cassette between US34 and TRS1
TB40/E-US28Flag	US28 gene contains 3 tandem Flag tags at carboxy terminus
TB40/E-ΔUS28	US28 gene deletion
TB40/E-ΔALL	UL33, UL78, US27, and US28 gene deletions

**Figure 1:**
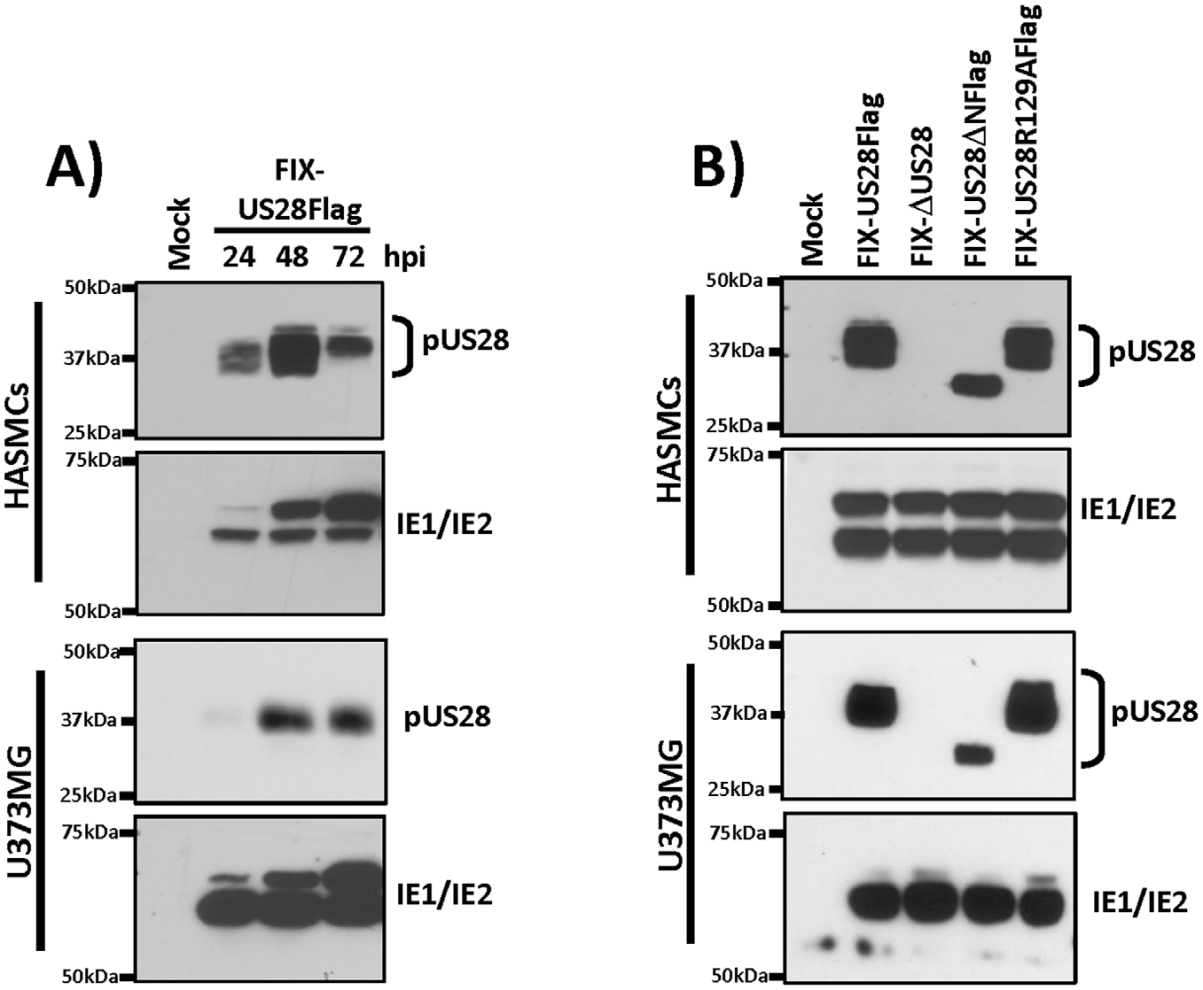
pUS28 is expressed in smooth muscle and glioblastoma cells. A) HASMCs and U373MG were infected with FIX-US28Flag at a MOI of 3 and pUS28 expression was analyzed by immunoprecipitation/western blot at the indicated times post-infection (upper panels). IE1/IE2 expression was analyzed by western blot and is shown for reference (lower panels). B) HASMCs and U373MG were infected with the indicated viruses at a MOI of 3 and pUS28 expression was analyzed by immunoprecipitation/western blot (upper panels) and IE1/IE2 expression was analyzed by western blot (lower panels). In each case, pUS28 expression was detected by IP for the Flag epitope, followed by immunoblot with a Flag antibody.

We initially analyzed the magnitude and timing of pUS28 expression during acute HCMV infection of human primary coronary artery smooth muscle cells (HASMC) and U373MG glioblastoma cells. Cells were infected with FIX-US28Flag at a multiplicity of infection (MOI) of three, extracts were prepared at time points between 24 and 72 hours post-infection (hpi) and pUS28 expression was analyzed by immunoprecipitation/western blot (Figure 1A). We also monitored IE1/IE2 protein levels at each time point to control for the HCMV infection process. In acutely infected HASMCs, pUS28 is robustly expressed and detectable at all the time points examined. In acutely infected U373MG cells, pUS28 is also expressed throughout the 72 hpi time course, albeit weakly at 24 hpi. For both HASMCs and U373MG cells, pUS28 expression reaches maximal expression at 48 hours hpi, and thus we chose to perform all additional experiments in HASMCs and U373MG cells at this time point. Next, we examined the relative expression of Flag tagged US28 wildtype, ΔN, and R129A mutant US28 proteins during HASMC and U373MG infection with the respective FIX-based recombinant viruses (Figure 1B). In both cell types, each of the US28 proteins was expressed to similar levels, although the ΔN mutant appeared to run on SDS-PAGE gels as a homogenous single band, while the wild-type and R129A variants of pUS28 appeared more as heterogeneous doublets. This was not examined further, but is likely the result of defective O-linked glycosylation by the ΔN mutant, as proposed by Casarosa, et al [33].

### US28 Constitutively Activates PLC-β in a Ligand-independent Manner in Smooth Muscle and Glioblastoma Cells

**Figure 2:**
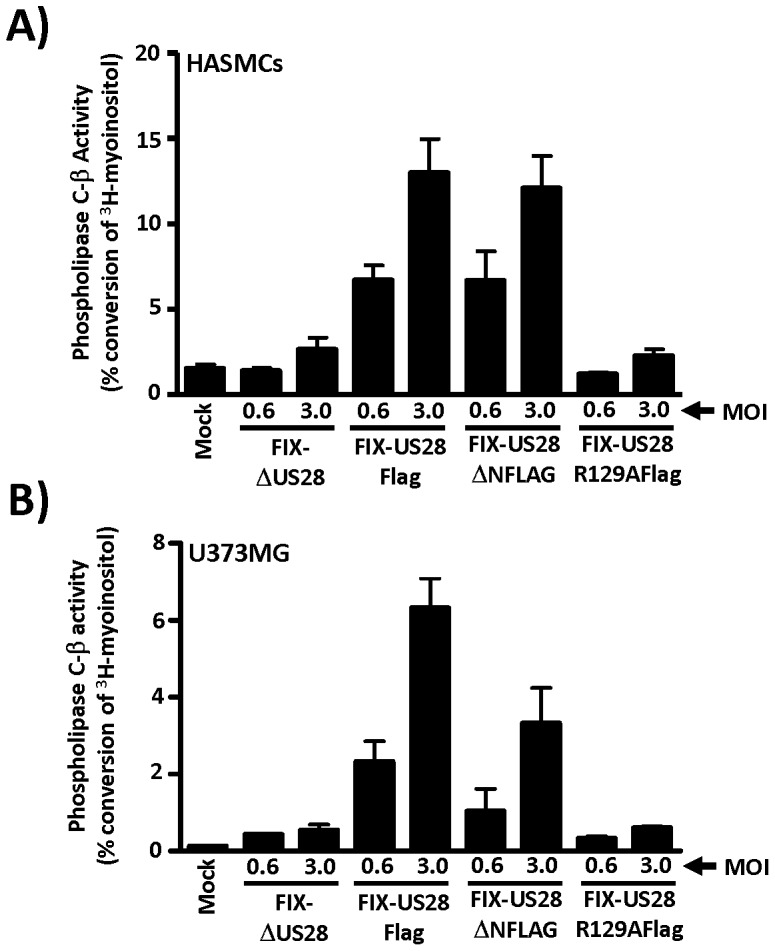
US28 constitutively activates PLC-β in a ligand independent but G-protein coupling dependent manner in HCMV infected smooth muscle and glioblastoma cells. A) HASMCs and B) U373MG were infected with FIX-US28Flag and each of the US28 mutant viruses at MOIs of 0.6 and 3.0. PLC-β activation was measured by labeling cells 24 hpi with 1 µCi/ml 3H-myoinositol followed by isolation of total inositol phosphates at 48 hpi. PLC-β activity is represented as the percent conversion of input myoinositol into inositol phosphates. The data presented are the results from at least three independent experiments performed in duplicate. The results are presented graphically and represent the mean+/−S.E. of four independent experiments performed in duplicate.

Given that US28 is predominantly a Gαq coupled GPCR, we chose to examine US28 signaling initially by assessing US28-promoted phospholipase C-β (PLC-β) activity. HASMCs and U373MG cells were infected with each of the FIX recombinant viruses (US28Flag, ΔUS28, etc) at MOIs of 0.6 to 3.0 and PLC-β signaling was assessed at 48 hpi, by measuring the accumulation of total inositol phosphates (InsP). The US28Flag and US28ΔNFLAG viruses strongly activated PLC-β signaling in the HASMCs (Figure 2A) and U373MG (Figure 2B) cells, while the ΔUS28 and US28R129A viruses failed to induce any measurable signaling through this pathway. These results indicate that US28 does in fact function as an active signaling molecule in HCMV infected smooth muscle and glioblastoma cells. Also, the data indicate that this PLC-β signaling occurs in an agonist-independent manner (as the ΔN mutant signals similar to wild-type) and that this signaling relies on G-protein coupling (as the R129A mutant fails to induce any activity). Moreover, we found that CCL5/RANTES had no effect on US28-driven PLC-β signaling confirming that this signaling pathway is strictly modulated by the agonist-independent signaling activity of US28 (Figure S1).

### US28 Interacting Chemokines CCL5/RANTES and CX3CL1/Fractalkine Induce Ca^++^ Production in HASMCs but not in U373MG Cells

**Figure 3:**
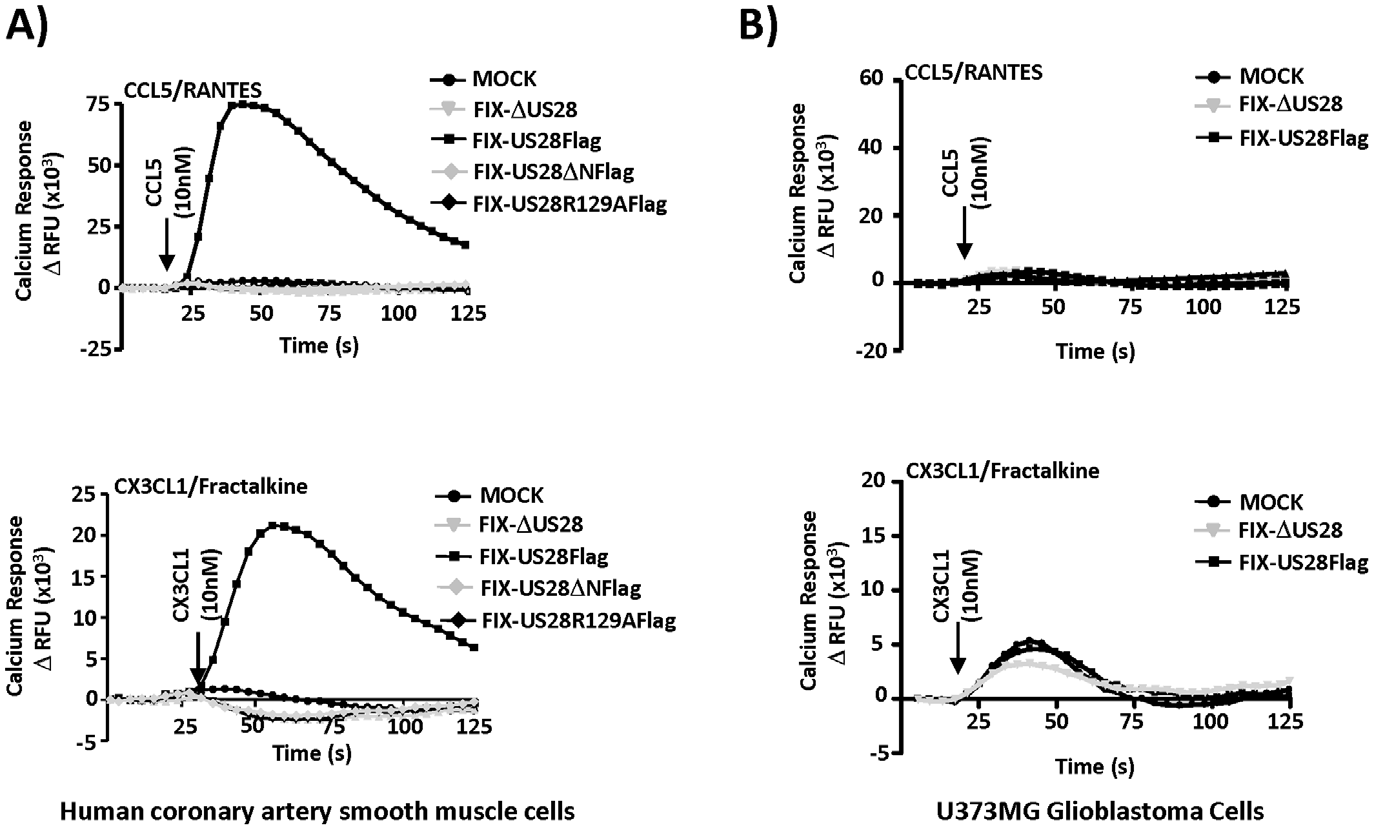
CCL5/RANTES or CX3CL1/Fractalkine binding to US28 triggers calcium release in HCMV infected smooth muscle but not glioblastoma cells. A) HASMC or B) U373MG cells were infected with the indicated FIX-based viruses at a MOI of 3. At 48 hpi, cells were labeled with Fluo-4 AM and stimulated with 10 nM CCL5/RANTES (top panels) or 10 nM CX3CL1/Fractalkine (bottom panels). Calcium responses were measured using a FlexStation II fluorometer. The calcium traces represent change in relative fluorescence units (ΔRFU) for each condition tested and are representative of at least three independent experiments performed in duplicate.

Vomaske and colleagues reported that CCL5/RANTES and CX3CL1/Fractalkine induce differential responses through US28 in smooth muscle cells infected with adenoviruses expressing US28 [39]. In their transient expression studies, CCL5 induced signaling through Gα12 and CX3CL1 inducing signaling through Gαq. However, in earlier studies from our laboratory, we demonstrated that both CCL5 and CX3CL1 could stimulate US28 and promote Ca^++^ release through a Gαq dependent pathway in HCMV infected fibroblasts [38]. Therefore, we investigated how these chemokines would affect US28 directed Ca^++^ signaling in smooth muscle and glioblastoma cells, in the context of viral infection. HASMCs were again infected with the FIX-based recombinant viruses and analyzed for CCL5 and CX3CL1 driven calcium release at 48 hpi. Interestingly, in smooth muscle cells, both of these US28 interacting chemokines were quite potent at stimulating US28 directed calcium mobilization, a pathway typically associated with Gαq/PLC-β/IP_3_ signaling (Figure 3A). Smooth muscle cells infected with the virus expressing wild-type Flag tagged US28 (FIX-US28Flag) responded to CCL5 (Figure 3A, upper panel) and CX3CL1 (Figure 3A, lower panel) with a rapid and robust change in intracellular calcium. HASMCs infected with viruses expressing US28ΔN (FIX-US28ΔNFlag) or US28R129A (FIX-US28R129AFlag) failed to respond to either chemokine. Note that while US28ΔN is capable of inducing strong constitutive signaling (Figure 2A), it is unable to mediate agonist-dependent Ca^++^ signaling (Figure 3A) consistent with pharmacological studies indicating that CCL5 or CX3CL1 binds to US28 via interaction with amino acids at the very amino terminus of US28. HCMV and US28 modulation of intracellular Ca^++^ was also plotted as raw fluorescence (Figure S2), demonstrating that HCMV infection itself causes an increase in intracellular calcium likely due to UL37x1 as reported by Shenk and colleagues [49] and that US28 can cause a further, but more transient change in intracellular calcium that requires the addition of extracellular agonists such as RANTES.

Surprisingly, while US28 exhibits strong agonist-independent signaling activity in the U373MG glioblastoma cells, we were unable to detect any agonist-dependent Ca^++^ signaling through US28 (Figure 3B). We did detect a low level Ca^++^ flux in response to CX3CL1/Fractalkine, although this signal was independent of US28 or HCMV as it was detected in both mock and HCMV infected cells (Figure 3B). It remains unclear why US28 would exhibit agonist-regulatable Ca^++^ signaling in one cell type (smooth muscle) but not in another (glioblastoma). pUS28 expression level and constitutive signaling are similar and therefore not likely to explain the failure to respond to agonist in the GBM cells.

**Figure 4:**
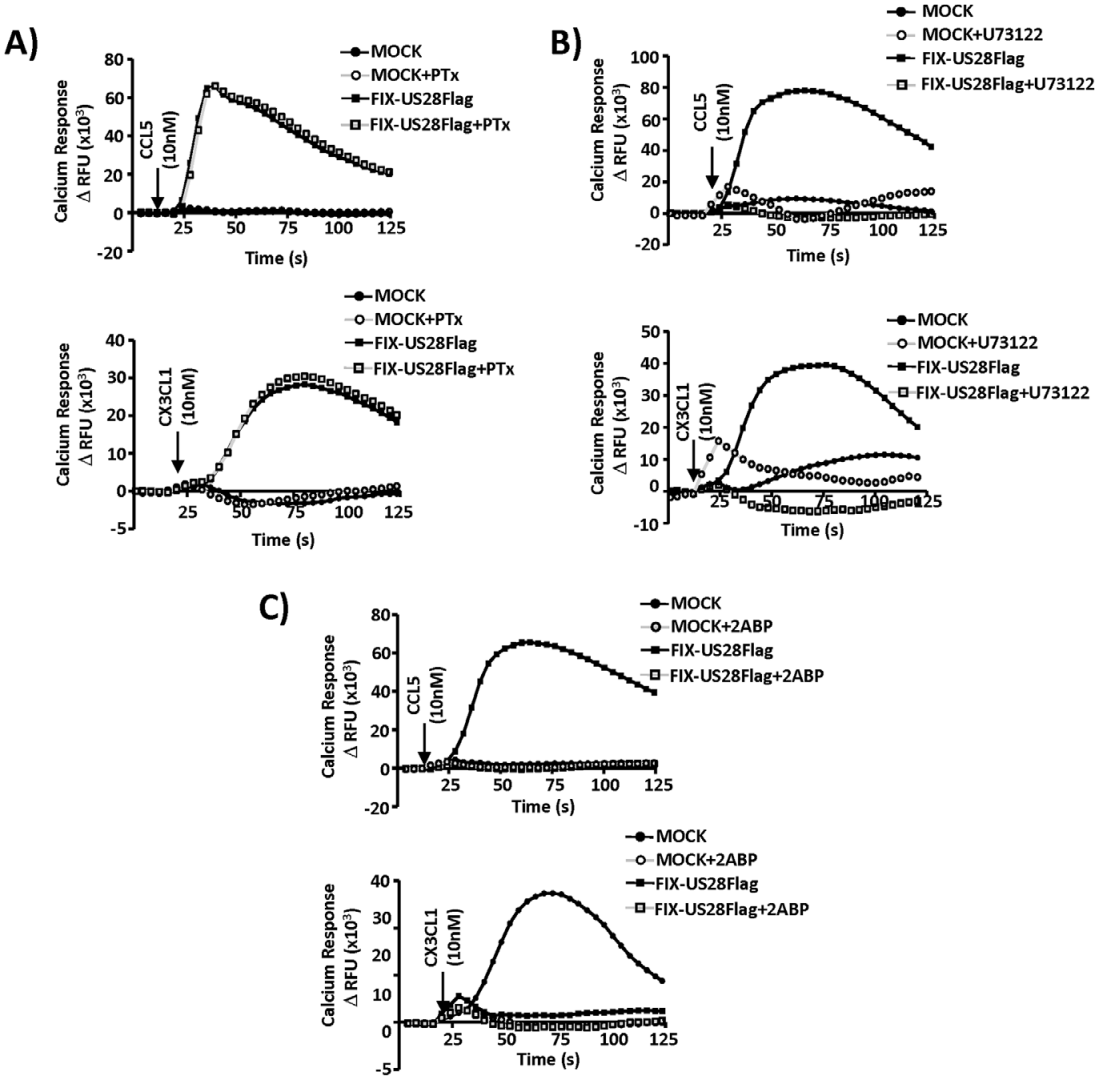
US28-mediated calcium release in infected HASMCs is activated by PTx insensitive G proteins, PLC-β, and IP_3_. To gain further insight into the agonist effects of CCL5 and CX3CL1 on US28-promoted Ca^++^ release, HASMCs were MOCK infected or infected with FIX-US28Flag virus. At 48 hpi cells were labeled with Fluo-4AM, treated with the Gαi inhibitor PTx (A), the PLC-β inhibitor U73122 (B), or the IP_3_ channel inhibitor 2ABP (C) and stimulated with 10 nM CCL5 or CX3CL1. Calcium responses were measured using a FlexStation fluorometer. The calcium traces represent change in relative fluorescence units (ΔRFU) for each condition tested and are representative of at least three independent experiments performed in duplicate.

To gain further insight into the mechanism of agonist-stimulated/US28-directed mobilization of intracellular Ca^++^ in HASMCs, we pretreated these cells with the Gαi inhibitor pertussis toxin (PTx), the PLC-β inhibitor U73122, or the IP_3_ channel inhibitor 2ABP (Figure 4). We found that PTx was without effect (Figure 4A), while the PLC-β and IP_3_ channel inhibitors inhibited US28 dependent Ca^++^ mobilization in response to both CCL5 and CX3CL1 (Figures 4B and 4C). Taken together, these results strongly argue that US28 expression in HCMV infected smooth muscle cells activates a Gαq/PLC-β/IP_3_ pathway leading to the mobilization of intracellular Ca^++^. Although the modulation of intracellular calcium by HCMV is important for viral replication in fibroblasts [49], it remains unclear if the additional Ca^++^ mobilization mediated by interaction of chemokines with US28 could affect HCMV replication under some conditions.

### Constitutive Signaling via US28 Hypersensitizes Smooth Muscle Cell Responsiveness to Lysophosphatidic Acid (LPA)

**Figure 5:**
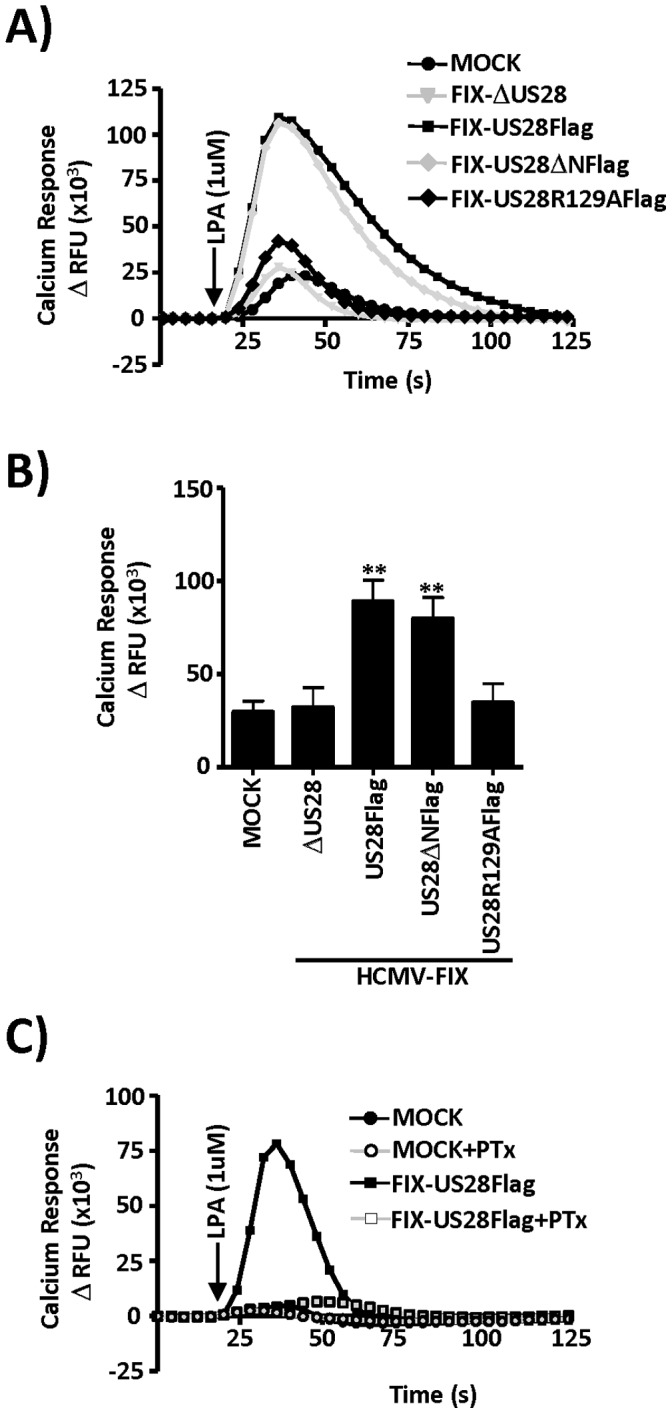
US28 potentiates signaling via endogenous Gαi coupled LPA receptors in HCMV infected arterial smooth muscle cells. A) HASMCs were infected with the indicated viruses at a MOI of 3. At 48 hpi, cells were labeled with Fluo-4 AM, stimulated with 1uM lysophosphatidic acid (LPA) and calcium flux was measured using a FlexStation II fluorometer. The results are also presented graphically (B) The results are presented graphically and represent the mean+/−S.E. of three to seven independent experiments performed in duplicate. C) To determine if the US28 effect was due to LPA signaling through endogenous Gαi proteins, we pretreated cells with the Gαi inhibitor PTx prior to stimulation with LPA. PTx completely abrogated the effects of US28 on LPA signaling indicating the involvement of Gαi in this process. The calcium traces represent change in relative fluorescence units (ΔRFU) for each condition tested and are representative of three to seven independent experiments performed in duplicate. **p<0.05..

US28 signaling in transiently transfected cells has been demonstrated to hypersensitize CCR1 to signaling via its cognate ligands, although this type of cross-talk between US28 and cellular GPCRs has not been investigated in HCMV infected cells [50]. We therefore hypothesized that pUS28 expression following HCMV infection may sensitize smooth muscle cells to respond more robustly to serum-derived ligands and chose lysophosphatidic acid (LPA) as the test case due to its abundance in serum and proximity to smooth muscle. Smooth muscle cell proliferation, survival, and chemotaxis are regulated to a large degree by active constituents of the serum such as LPA [51], [52], [53], [54]. LPA signals via a group of receptors within the GPCR superfamily termed LPA_1–6_, which signal primarily via the Gαi family of G-proteins. We infected HASMCs with our panel of FIX recombinant viruses and at 48 hpi examined the responsiveness of these cells to 1uM LPA. Cells expressing either wild-type Flag tagged US28 (FIX-US28Flag) or N-terminally truncated US28 (FIX-US28ΔNFlag) responded very strongly to LPA, while uninfected cells, cells infected with the US28 null virus (FIX-ΔUS28), or cells infected with the G-protein uncoupled US28 (FIX-US28R129A) virus responded only weakly to LPA (Figure 5A). The results of several independent experiments are also presented graphically (Figure 5B). To determine if the US28 effect was due to LPA signaling through endogenous Gαi proteins or if the observed effects could be the result of LPA binding to US28 and signaling via Gαq, we pretreated cells with the Gαi inhibitor PTx prior to stimulation with LPA. PTx completely abrogated the effects of US28 on LPA signaling indicating the involvement of Gαi in this process (Figure 5C). Our data suggests that constitutive signaling via US28 can integrate or “fine tune” signal transmission by initiating cross-talk between multiple classes of GPCRs similar to that observed for other cellular Gαq coupled receptors.

### US28 is a Potent Signaling Molecule in GBM Cells in the Absence of Strong Expression of the Protein

**Figure 6:**
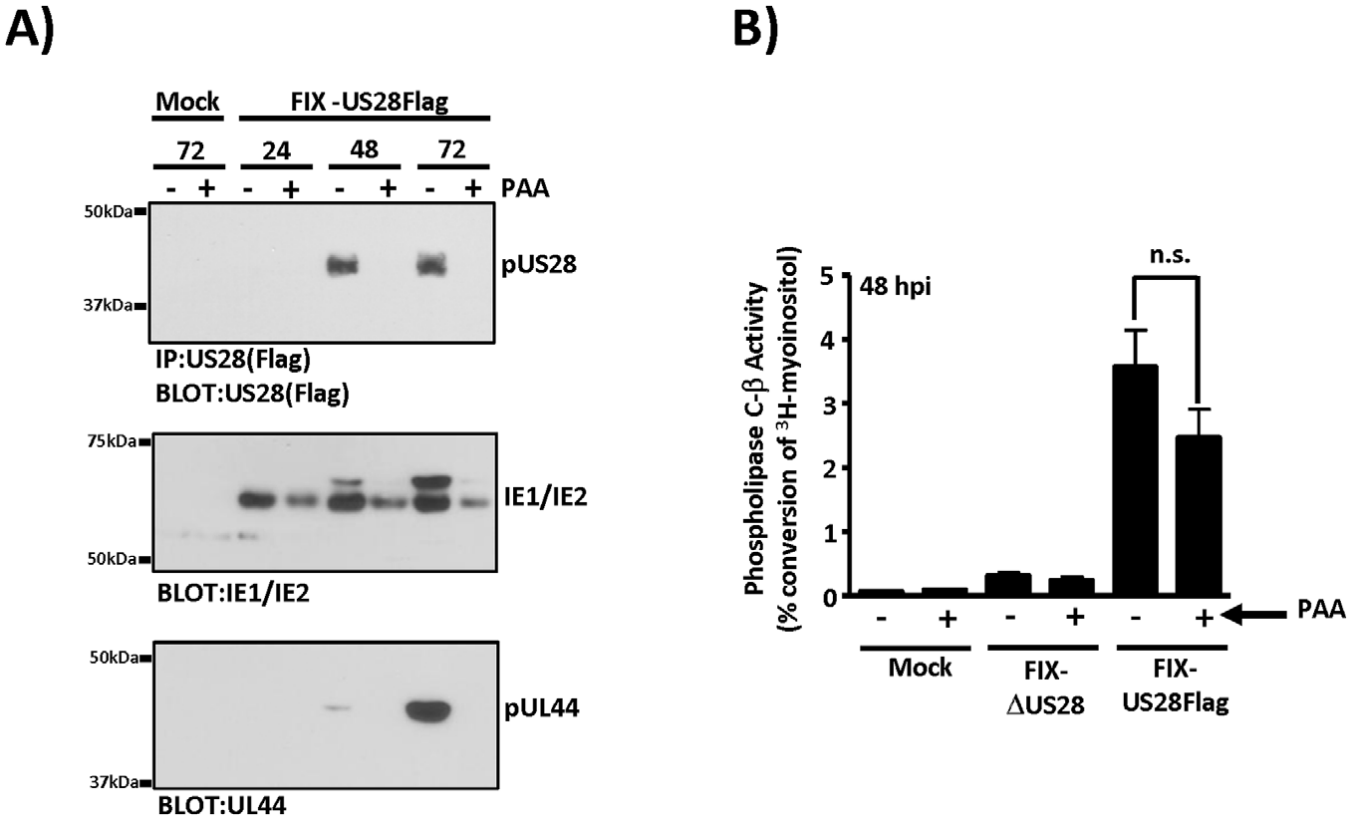
Inhibition of viral replication in HCMV infected glioblastoma cells severely restricts pUS28 expression but only modestly affects the total magnitude of US28 signaling activity. A) U373MG were mock infected or infected with FIX-US28Flag at a MOI of 3 and pUS28, IE1/IE2, and pUL44 expression was analyzed by immunoprecipitation/western blot (pUS28) or western blot (IE1/IE2 and pUL44) at the indicated times post-infection. To determine what effect inhibition of viral replication has on US28 expression in U373MG cells, cells were treated with or without the replication inhibitor phosphonoacetic acid (PAA) at a concentration of 2 mM, where indicated. B) US28 stimulated PLC-β activity was analyzed in the presence or absence of PAA. PLC-β activation was measured by labeling cells 24 hpi with 1 µCi/ml 3H-myoinositol followed by isolation of total inositol phosphates at 48 hpi. PLC-β activity is represented as the percent conversion of input myoinositol into inositol phosphates. The data presented are the results from three independent experiments performed in duplicate.

US28 is considered a delayed-early/late gene, as accumulation of US28 mRNA (as assessed by northern blot analysis) is largely inhibited by the viral polymerase inhibitor phosphonoacetic acid (PAA), and US28 protein reaches maximal levels at 48–72 hpi [35], [43], [55]. Thus, if US28 expression is largely restricted to late times in the lytic cycle it would seem unlikely that any growth-promoting or oncogenic effects of US28 would be manifested in cells destined to undergo cell death as a result of lytic replication of the virus. Therefore, we set out to examine US28 signaling in cells in which lytic replication was pharmacologically inhibited to determine if it is possible that US28 could be an active signaler under conditions of non-permissive viral infection. To determine to what extent PAA effects US28 protein expression, we infected U373MG cells at a MOI of 3 and treated the cells with or without 2mM PAA from the time of virus adsorption. Expression of pUS28 was analyzed at 24, 48, and 72 hpi by immunoprecipitation/western blot (Figure 6A, upper panel). Interestingly, pUS28 expression was essentially undetectable by immunoprecipitation/western blot in cells treated with the replication inhibitor. Expression of immediate early proteins (IE1/IE2) and a delayed-early protein that accumulates to high levels during the late phase (pUL44) [56], [57], [58] were also examined and confirmed that the PAA was functioning as expected (Figure 6A, middle and lower panels). Moreover, analysis of virus replication as assessed by plaque assay confirmed that HCMV replication was inhibited in the PAA treated cells (data not shown). As pUS28 reaches maximal expression at 48 hpi, we analyzed US28-dependent PLC-β signaling in U373MG cells treated with or without PAA (Figure 6B). As expected, US28 induced a robust agonist-independent PLC-β signal in agreement with western blot analysis of pUS28 expression. Surprisingly, in PAA treated cells in which US28 protein expression was undetectable by western blot, we observed strong (albeit slightly reduced) US28 driven PLC-β signaling (Figure 6B). These results confirm that the PLC-β signaling is wholly attributable to US28 as the mock and FIX-ΔUS28 infected cells exhibit only background levels of signaling (0.06% and 0.09% conversion, respectively) and that the PAA exhibited little to no effect on US28 signaling (2.5% versus 3.6% conversion, p = 0.1774). Taken together, these results suggest that US28 is a very potent signaling molecule, whose protein levels in the absence of viral genome amplification during lytic replication are enough to generate a strong signaling response. These findings provide a plausible avenue by which low-level US28 protein expression in non-lytically infected GBM cells could provide a signal sufficient to promote cellular growth.

### Analyses of US28 Signaling in a Tet-regulatable System Demonstrates Potency of US28 Signaling Activity

**Figure 7:**
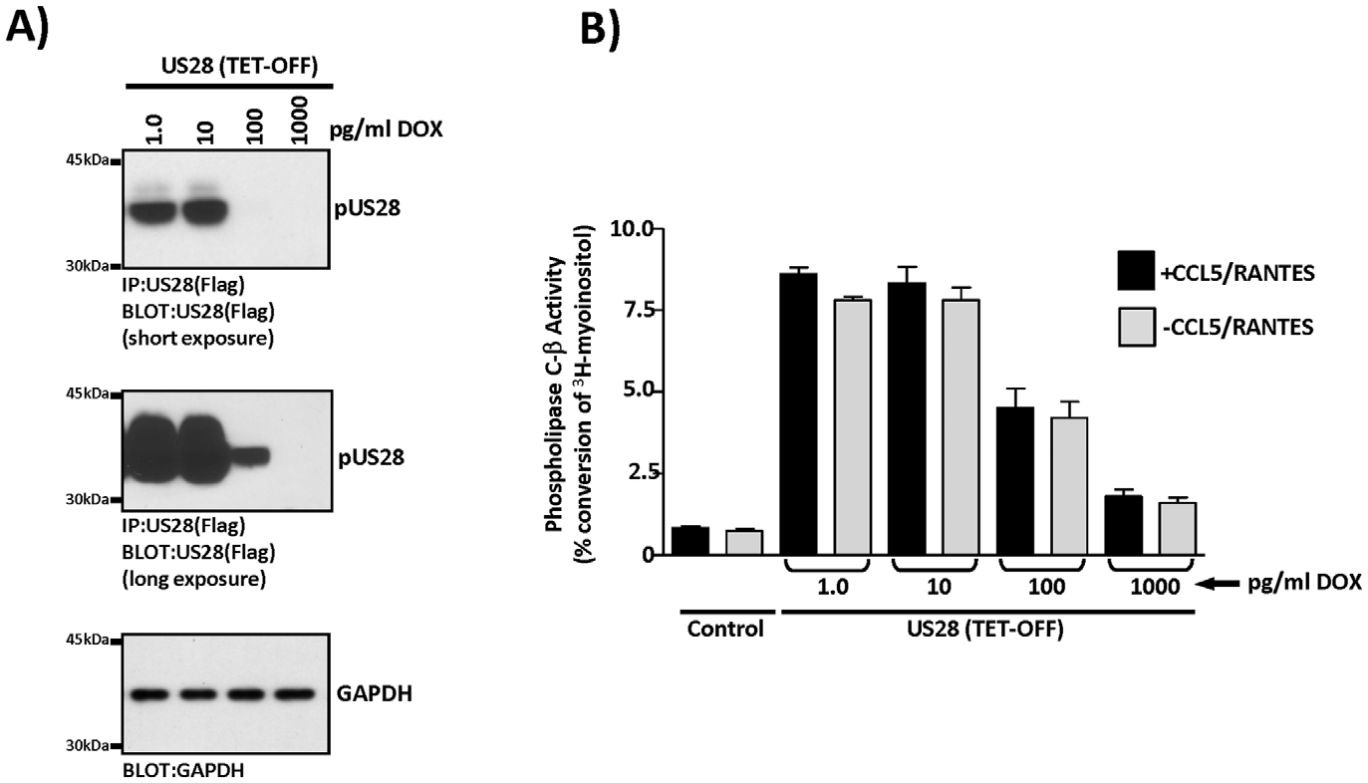
US28 is a potent activator of signaling even at very low expression levels. A) Development of a HEK-based Tet-regulatable US28 expression system and analysis of DOX regulated changes in US28 protein levels. US28 expression was titrated using DOX concentrations ranging from 1 pg/ml to 1000 pg/ml. pUS28 expression was analyzed by western blot and the results indicate that 100 pg/ml DOX blocks US28 expression >90%, while 1000 pg/ml DOX blocks US28 expression >99%. Cellular GAPDH expression in whole cell lysates is shown for control purposes. B) US28 stimulated PLC-β activity was then analyzed over the range of pUS28 expression levels. Interestingly, while 100 pg/ml DOX blocks pUS28 expression >90%, the corresponding drop in US28 stimulated PLC-β was ∼45%. Similarly, while 1000 pg/ml blocks pUS28 expression >99%, the corresponding drop in US28 stimulated PLC-β was ∼75%. CCL5/RANTES was used at a concentration of 10 nM.

Our experiments in U373MG glioblastoma cells treated with PAA to pharmacologically inhibit HCMV replication suggest that pUS28 is an extremely potent signaling molecule as the PAA reduces pUS28 expression to undetectable levels, but minimally affects US28 signaling activity. To further explore this postulate, we established a TET-regulatable system in which we can carefully regulate the production of US28 protein and evaluate the ensuing level of US28-dependent signaling. We stably transfected HEK/Tet-off cells with a TET-regulatable US28 expression construct and a representative clone exhibiting tight DOX regulation following sensitivity testing was chosen for further analysis (data not shown). To evaluate the level of control that DOX has on pUS28 expression in this system, we treated cells with increasing concentrations of DOX (1–1000 pg/ml) and evaluated pUS28 expression by immunoprecipitation/western blot (Figure 7A). This system exhibits tight control, such that pUS28 expression remains at 100% at 10 pg/ml DOX, but drops to less than 10% at 100 pg/ml DOX. Multiple exposures of the western blots are shown to demonstrate the effects of DOX in this system (Figure 7A, upper and middle panels). We then evaluated US28 stimulated PLC-β signaling activity at the various DOX controlled levels of pUS28 expression (Figure 7B). At maximal levels of expression (1.0 and 10 pg/ml DOX), US28 robustly stimulated PLC-β activity (7.8% conversion at both DOX levels), while at very low levels of pUS28 expression (100 pg/ml DOX), US28 stimulated PLC-β activity remained strong (4.2% conversion). Thus, while 100 pg/ml DOX reduced pUS28 expression to levels less than 10% of maximal, its signaling activity was only reduced about 45% (4.2% conversion compared to 7.8% conversion). Taken together with studies in PAA treated U373MG glioblastoma cells, our data argue that US28 is a very potent constitutive signaler and is likely to exhibit significant cellular effects even at very low expression levels.

Interestingly, the effect of CCL5/RANTES on US28 stimulated PLC-β signaling is negligible regardless of the absolute level of pUS28 expression (Figure 7B). There has been some question as to whether the constitutive signaling of US28 through PLC-β is a consequence of high expression levels and perhaps US28 signaling through PLC-β occurs in an agonist-dependent fashion when pUS28 is expressed at low levels. However, the results presented here conclusively demonstrate that the relative fraction of agonist-independent constitutive PLC-β signaling activity of US28 does not vary depending on pUS28 expression levels.

### US28 Exhibits Agonist-independent Signaling in HCMV Infected Endothelial Cells, but does not Affect Viral Gene Expression or Replication Efficiency

**Figure 8:**
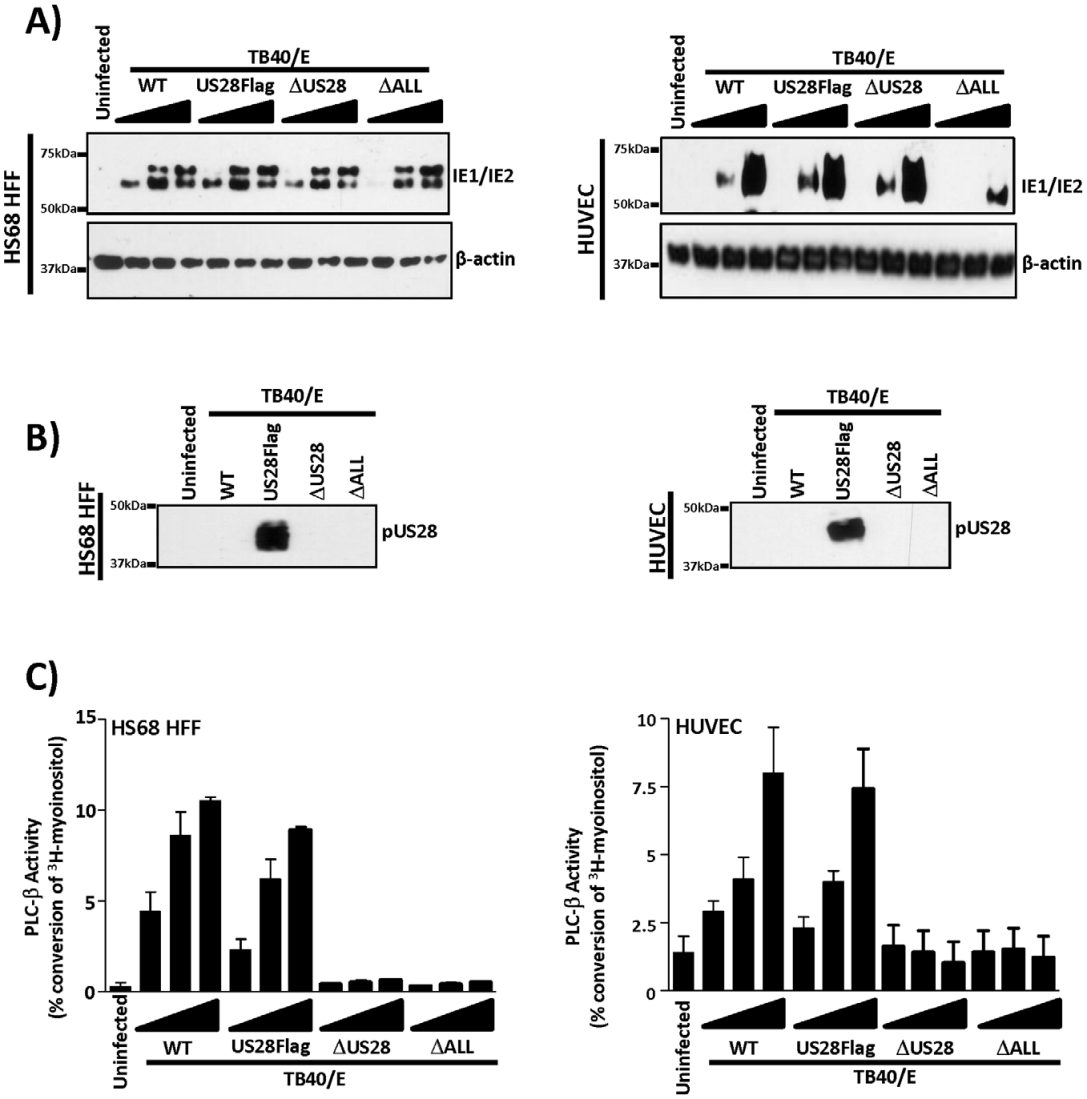
US28 expression and PLC-β signaling in fibroblasts and endothelial cells infected with TB40/E recombinant viruses. A) HS68 HFFs infected at MOIs of 0.2, 1, and 5 (left panel) or HUVECs infected at MOIs of 0.6, 3, and 15 (right panel) were analyzed by western blot for IE1/2 or β-actin expression at 48 hpi. B) Extracts prepared from HFFs infected at a MOI of 5 (left panel) or HUVECs infected at a MOI of 15 (right panel) were analyzed by immunoprecipitation/western blot for pUS28 expression at 48 hpi. C) HS68 HFFs (left panel) or HUVECs (right panel) infected with increasing MOIs as described in panel A were analyzed for PLC-β activity at 48 hpi. In each cell type, PLC-β activation was measured by labeling cells 24 hpi with 1 µCi/ml 3H-myoinositol followed by isolation of total inositol phosphates at 48 hpi. PLC-β activity is represented as the percent conversion of input myoinositol into inositol phosphates.

Endothelial cells represent an important site for viral replication *in vivo* and are unique in that HCMV infection and replication in these cells requires a select set of viral genes that are dispensable for replication in most other cell types. Since US28 is a potent signaling molecule when expressed in other cell types (fibroblasts, smooth muscle, etc), it is important to examine US28 expression, signaling and influence on viral replication in acutely infected endothelial cells. The FIX derivatives used in other experiments in this study were not amenable for endothelial cell studies as we were not able to achieve infection rates sufficient for the signaling studies with US28 (data not shown). Furthermore, although FIX infects a broad range of cell types relevant to *in vivo* infection, this seems to be at the cost of robust viral titers. BAC-derived TB40/E, like the FIX strain, is a clinical isolate that grows to significantly higher titers compared to FIX (i.e.: 2-logs in fibroblasts; 3-logs in endothelial cells) [59], while maintaining broad cell tropism [59], [60], [61], [62], [63]. Although it is currently unclear as to the factors that contribute to the varied growth phenotypes, it is clear that BAC-derived TB40/E is a useful tool for investigating HCMV in broad cell types. Therefore, we turned to newly constructed TB40/E-based viruses in which we have inserted three tandem Flag epitope tags into US28 (US28Flag), deleted US28 (ΔUS28), or deleted all four HCMV-encoded GPCRs (ΔALL) (Table 1). In constructing these new variants, we chose a tandem epitope tag, as this is a more sensitive means by which to detect intracellular protein localization and expression, and will serve as a useful tool for further investigations. We performed an initial characterization of these new viruses in fibroblasts and examined IE1/2 expression (Figure 8A, left panel), pUS28 expression (Figure 8B, left panel) and PLC-β signaling at 48 hpi (Figure 8C, left panel). Similar to our earlier studies in fibroblasts, we find that TB40/E based recombinants robustly express pUS28 and lead to high levels of US28-promoted PLC-β activity [38], [43].

Following this initial characterization in fibroblasts, we performed a similar analysis in endothelial cells. Since serially passaged HCMV stocks have a tendency to lose their ability to infect endothelial cells, infection was carefully monitored by evaluating mCherry expression using flow cytometry (Figure S3). In general, higher MOIs are necessary for HUVECs to support robust viral infection, and as such we achieved consistent infection rates with each recombinant virus, approaching ∼75% when an MOI of 15 is used. Examination of IE1/IE2 protein levels at 48 hpi indicated that the wild-type, US28Flag, and ΔUS28 viruses similarly entered into the immediate-early phase, while the ΔALL mutant exhibited a reduced ability to enter into the immediate-early phase (Figure 8A, right panel). We will discuss this apparent defect in HCMV immediate early gene expression with the ΔALL mutant later in this manuscript.

We then analyzed pUS28 expression and signaling in HUVECs acutely infected with our TB40/E-based mutants. Similar to our findings in HASMC, GBM, and HFF cells, immunoprecipitation/western blot analyses indicated that pUS28 is strongly expressed at 48 hpi in HUVECs (Figure 8B, right panel) and therefore we carried out experiments at this time point to determine if US28 is actively signaling. HUVECs infected with the TB40/E-based mutants were labeled with ^3^H-myoinositol and PLC-β activity was measured. Similar to our results in the other cell types tested, we found that US28 regulates PLC-β signaling activity in a constitutive manner as the TB40E-ΔUS28 and TB40E-ΔALL mutants failed to alter PLC-β activity (Figure 8C, right panel). Moreover, as the ΔUS28 and ΔALL mutants are similarly defective in PLC-β activity, the results indicate that US28 is solely responsible for PLC-β signaling in the context of HCMV infection. Taken together, our results indicate that US28 is ubiquitously expressed and exhibits constitutive signaling in acutely infected cells, regardless of the cellular context.

**Figure 9:**
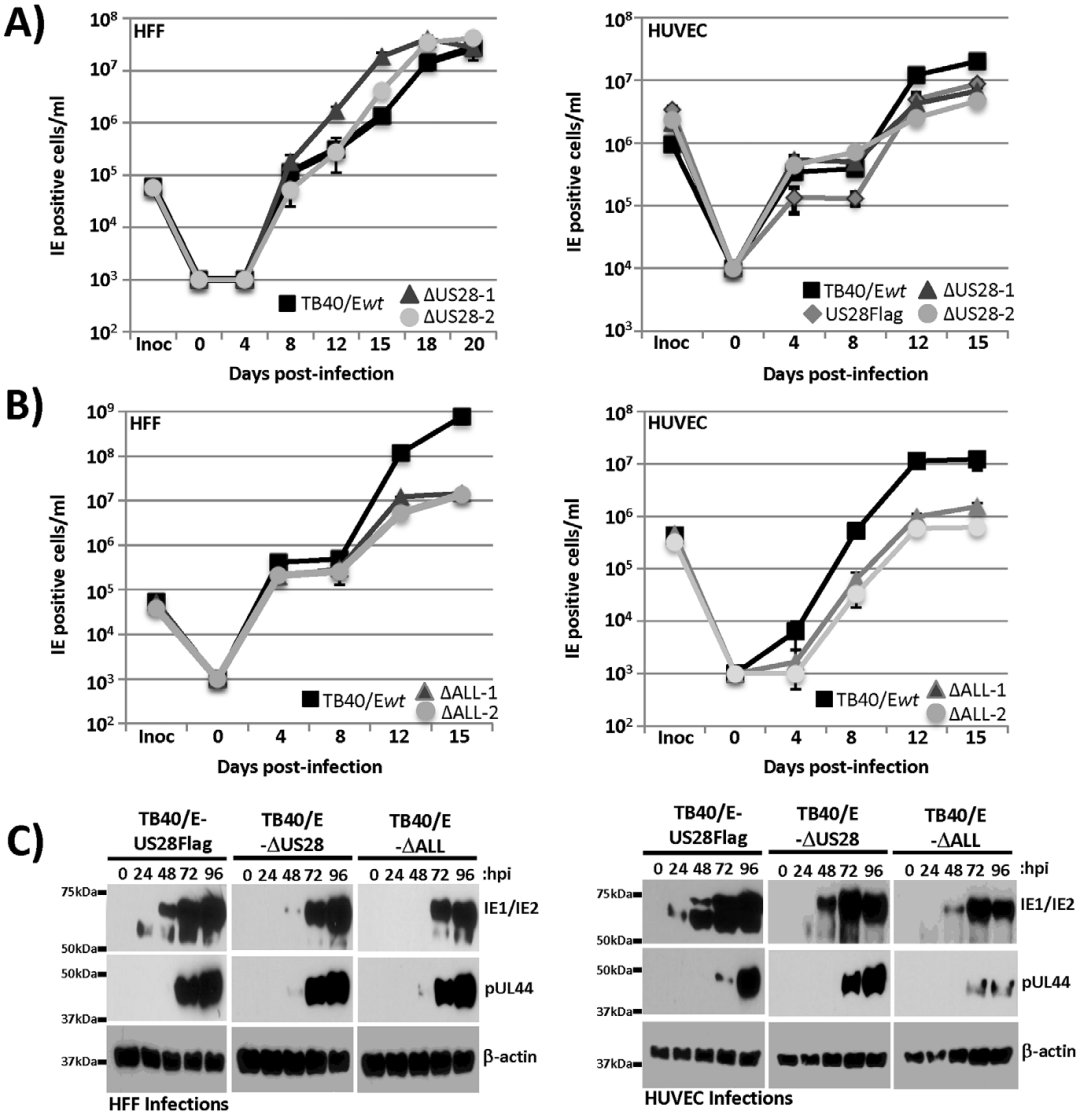
Viral replication in fibroblasts and endothelial cells infected with TB40/E recombinant viruses. A) To examine growth kinetics of wild-type and ΔUS28 viruses, HFFs (left panel, MOI = 0.01) or HUVECs (right panel, MOI = 0.1) were infected with TB40/E*wt*-mCherry or the ΔUS28 recombinants, samples of tissue culture supernatant were collected at the indicated time points, and viral progeny was assayed by infecting fibroblasts and quantifying IE1-positive cells 24 h later by immunofluorescence. B) To examine growth kinetics of wild-type and ΔALL viruses, HFFs (left panel, MOI = 0.01) and HUVECs (right panel, MOI = 0.1) were infected with TB40/E*wt*-mCherry or the ΔALL recombinants and viral progeny was assayed as in panel A. C) HFFs infected at a MOI of 3 (left panels) or HUVECs infected with an MOI of 15 (right panels) were analyzed by western blot for IE1/2, pUL44 or β-actin expression over a 96 hour time course following infection.

Since US28 exhibits a strong propensity to initiate PLC-β signaling in the endothelial cells and since a number of HCMV genes have been demonstrated to be important for HCMV replication in HUVECs, we then tested the ability of these newly derived ΔUS28 and ΔALL viruses to replicate and produce infectious virus in endothelial cells (HUVECs). Similar to previously published reports, viruses deleted for US28 (TB40E-ΔUS28) exhibited no defect in the kinetics of viral gene expression or replication in fibroblasts (HFFs) as they grew to similar virus titers as their wild-type derivatives (Figures 9A, left panel) and they similarly expressed IE and E proteins (Figure 9C, left panel) [21], [22], [35]. Moreover in HUVECs, the TB40E-ΔUS28 virus exhibited no defect in replication (Figure 9A, right panel) or viral gene expression (Figure 9C, right panel). Thus US28 itself does not appear to be essential for viral replication in cell culture regardless of the cell type tested.

With four GPCRs present in the HCMV genome (UL33, UL78, US27, US28) questions regarding redundancy become important. In other words, is there a biological activity emanating from these GPCRs that is important for replication *in vitro*, but not readily apparent due to the presence of multiple GPCR genes? Therefore, we tested the ability of the TB40/E-ΔALL virus to proceed through the lytic cycle and replicate in HFFs and HUVECs. The TB40/E-ΔALL virus exhibits a moderate replication defect (1–2 logs) in both HFFs and HUVECs (Figures 9A and 9B, right panels). In HUVECs, the TB40E-ΔALL virus does fail to efficiently proceed through the immediate-early and early phases of gene expression (Figure 8A, right panel and Figure 9C, right panel) thereby providing a potential explanation for the replication defect in this cell type, although we did not observe the same defect in viral gene expression in HFFs. This is consistent with our earlier work, demonstrating the deletion of US27 results in decreased extracellular viral spread in fibroblasts and endothelial cells, where at least in fibroblasts, the accumulation of representative IE, E, and L proteins are similar to wild-type [44]. Additionally, deleting the UL78 ORF results in reduced accumulation of IE1, pUL44, and pUL99 in endothelial cells [60]. We are currently investigating the possibility that the phenotypes we observed in viral protein accumulation and viral growth are additive when multiple GPCR ORFs are deleted in a single background.

## Discussion

Our knowledge regarding the functional significance of the human cytomegalovirus (HCMV) encoded G-protein coupled receptor (GPCR) family has been hampered by several key factors. First, these genes are not essential for viral replication *in vitro* in commonly used cell culture models. Second, studies aimed at determining how these genes facilitate viral replication and pathogenesis *in vivo* has been prevented by cross-species constraints exhibited by members of the cytomegalovirus family. Third, the complexities of virus infection experiments make it difficult to examine molecular signaling activities emanating from these viral encoded GPCRs in cells infected with HCMV (i.e., GPCR-dependent signals must be measured at times corresponding to their expression windows while concurrently accounting for the possibility that the onset of lytic replication and the induction of cellular death can dramatically affect the measurement of these GPCR signals). Finally, since most serially passaged/laboratory-adapted HCMV strains have lost their endotheliotropic properties, recombinant viruses with mutations in viral GPCR genes must be made in the appropriate clinical strain background before any analyses of signaling can be performed in endothelial cells.

Overexpression type approaches such as transient/stable transfections, adenoviral infection, and generation of transgenic mice have, however, revealed a number of interesting molecular and biological properties of viral GPCRs like US28. The basic molecular signaling activities, as revealed from these approaches, include constitutive activation of Gαq/PLC-β signaling, agonist-dependent stimulation of Ca^++^ release, and agonist-dependent regulation of a Gα12 signaling pathway leading to activation of Src, FAK, and Rho. From a biological perspective, these overexpression approaches have demonstrated that US28 is involved in cellular proliferation and stimulation of cellular migration. Ongoing work aimed at integrating the molecular signaling and biological activities of US28 is likely to provide important insight into the role that GPCRs like US28 play in pathogenesis. There is some data with HCMV US28 mutants indicating that US28 can stimulate smooth muscle cell migration in the context of HCMV infection, however this data is limited and most of the studies in this regard have utilized adenovirus or transient transfection approaches [20], [23], [24], [39].

Studies using rodent models of CMV pathogenesis have revealed essential *in vivo* roles for proteins like M33, R33, M78, and R78; however the species barrier has prevented studies aimed at determining if the HCMV counterparts like UL33 and UL78 play similar roles *in vivo*. Moreover, genes for US27 and US28 are not present in rodent CMV genomes and therefore one cannot even extrapolate as to what the functions of these genes might be from rodent models.

**Figure 10:**
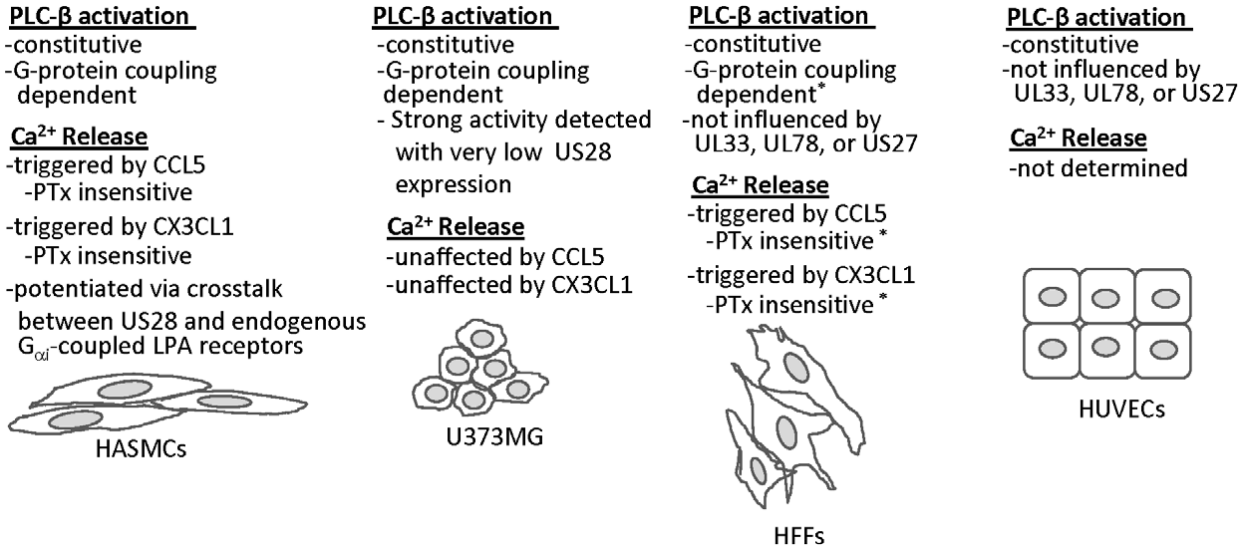
Summary of findings. A summary of our findings in all cell types tested, included smooth muscle cells (HASMCs), GBM cells (U373MG), fibroblasts (HFFs), and endothelial cells (HUVECs). *Ca^++^ data and G-protein dependency in HFF cells was determined previously and published in Stropes et al. [38].

In the studies reported here, we have assessed the timing and expression of US28 during HCMV infection of a number of clinically important cell types and discovered that US28 constitutively activates the PLC-β/IP_3_ signaling pathway in all cell types tested including smooth muscle, endothelial, and glioblastoma (Summarized in Figure 10). Thus, it appears that this potent and agonist-independent signaling pathway is activated in all HCMV-infected cells and occurs without the influence of the cellular environment or presence of cytokines/chemokines in the extracellular milieu. The downstream ramifications of this constitutively active PLC-β/IP_3_ signaling are widespread and it is for this reason that we focused on the most proximal readout for this signaling pathway (measurement of PLC-β stimulated IP_3_ accumulation). Investigators have reported that numerous signal transducers downstream of the PLC-β signaling pathway are turned on in cells transiently expressing US28 including NF-κB, NFAT, CREB, VEGF, COX-2, IL6/STAT3, etc [15], [19], [64], [65]. A determination of which of these downstream pathways is activated in each cell type is beyond the reach of the current study, however, our data demonstrates for the first time that the proximal signalers in this pathway (e.g. Gαq/PLC-β) are activated in numerous cell types infected with HCMV. We were unable to demonstrate agonist-dependent activation of the Gα12/Rho pathway in HCMV-infected cells using our recombinant clinical isolates (data not shown); however, we were able to demonstrate a cell-specific agonist-dependent regulation of Ca^++^ release. We detected high level US28-specific release of Ca^++^ in response to CCL5/RANTES or CX3CL1/Fractalkine in primary smooth muscle cells but not in the glioblastoma derived cells, although it is currently unclear why US28 would fail to induce this response in the glioblastoma cells. Since US28 is constitutively active in the glioblastoma cells and the agonists are added exogenously, it appears that some step in the pathway downstream of PLC-β/IP_3_ is not active in the glioblastoma cells. This finding highlights the potential diversity of the US28 signal depending on the cell type in which it is expressed.

It is interesting that both the constitutive PLC-β→IP_3_ accumulation and the agonist-dependent PLC-β→IP_3_→Ca^++^ release in smooth muscle cells appears to proceed through a similar pathway involving PLC-β and IP_3_ accumulation activation. These findings are entirely consistent with earlier publications, which demonstrate that US28 is a constitutive inducer of IP_3_ accumulation, yet promotes Ca^++^ only in the presence of ligand [14], [15], [21], [35], [38]. It remains unclear if the magnitude of IP_3_ accumulation in the absence of agonist is insufficient to stimulate ER localized IP_3_ receptors and drive calcium release or if the US28 agonists activate another as yet unidentified signaling intermediate that allows ER localized IP_3_ receptors to respond to increased IP_3_. We are currently exploring these possibilities.

Since US28 expression in primary smooth muscle cells leads to both agonist-dependent and agonist-independent signaling, we surmised that this would be an excellent model in which to examine the effects of US28 on signaling via other cellular GPCRs expressed endogenously in HCMV-infected smooth muscle cells. US28 and other “constitutively” active Gαq coupled receptors have been reported to enable signaling via Gαi-coupled GPCRs present in the same cell [50]. To date this has not been examined in HCMV-infected cells and since the Gαi coupled LPA receptors have important biological effects in smooth muscle [51], [52], [53], [54], we explored the possibility that there might be cross-talk between US28 and LPA receptors. Interestingly, we detected cross-talk between these two signaling pathways, as the presence of US28 hypersensitizes the cells to LPA. These results demonstrate for the first time that constitutively active US28 signaling in infected smooth muscle cells impacts signaling via other receptors present in the same cell. While the biological implications vis-à-vis LPA and smooth muscle cell migration are significant, this finding represents a phenomenon that is likely to occur in any HCMV-infected cell depending on the expression of cellular Gαi coupled receptors and the corresponding availability of ligands for those receptors in the adjacent extracellular space. The mechanism by which this cross-talk occurs is currently unknown, although previous reports regarding cross-talk between US28 and CCR1 in transfected cells involved the activity of the heterotrimeric G-protein subunits Gβγ [50]. We are currently exploring the mechanism of signaling and impact that this crosstalk may have on viral pathogenesis.

This is the first study in which HCMV recombinants with altered US28 genes are used to molecularly dissect US28 signaling in HCMV-infected glioblastoma cells. Recent studies indicate a high prevalence of HCMV infection in GBM tumors. Although not typically considered an oncogenic virus, HCMV encodes several proteins including US28 that have been demonstrated to have oncogenic activities in transfected or transgenic settings [19], [25], [26]. Moreover, recent work from Cobb and colleagues suggests that US28 expression in GBM cells promotes an invasive and angiogenic phenotype [66]. Our studies with FIX-US28 recombinants provide essential information regarding constitutive and agonist-dependent signaling GBM cells *in vitro* in the context of HCMV infection and provide an essential foundation from which to eventually investigate the mechanism(s) by which US28 may facilitate the transformative process. Additionally, while it is clear that GBM cells such as U373MG support lytic replication of HCMV, virus production is extremely low. Therefore the U373MG cell system provides an opportunity to examine US28 signaling in a productive setting distinct from that of cells that support robust lytic replication like those derived from fibroblasts and smooth muscle. We found that US28 does activate the PLC-β signaling pathway in a constitutive manner in the glioblastoma cells, but fails to respond to extracellular ligands such as CCL5/RANTES and CX3CL1/Fractalkine to promote the release of intracellular calcium. Interestingly, we found that US28 is a very potent signaler, even when low levels of US28 protein are produced in cells treated with the viral replication inhibitor phosphonoacetic acid (PAA). We pursued this line of investigation for two reasons. First, US28 expression peaks at late times post-infection after the production of infectious virus begins and we sought to determine if US28 was expressed at levels sufficient to induce signaling under conditions in which nascent virions are not being produced. Second, lytic replication and cellular lysis in glioblastoma cells would seem inconsistent with tumor cell growth and thus we wished to exploit this system and ask how US28 would signal under non-permissive replication conditions in glioblastoma cells. We were surprised to find that PAA treatment of glioblastoma cells reduced expression of US28 protein to undetectable levels, but had little to no effect on US28 signaling. Thus, US28 is likely to have profound effects on cellular signaling even when expressed at low levels in non-permissive cells or even in latently infected cells such as CD34^+^ hematopoietic progenitors. Interestingly US28 mRNAs are expressed in several models of HCMV latency, so it will be interesting to determine if US28 signaling has an impact on HCMV latency/reactivation. We used an inducible Tet-regulatable system to provide independent confirmation that US28 is an extremely potent signaler even at low levels of protein expression. Using this system, we reduced the expression of US28 to barely detectable levels, yet retained 50–75% of maximal signaling activity exhibited by high US28 expression levels. Moreover, using the Tet-off system we were able to conclusively demonstrate that this agonist-independent signaling activity exhibited by US28 is not the result of low levels of leaky receptor activity that manifests itself as significant simply due to overexpression. We determined that the ratio of US28 signaling (+/− RANTES) is roughly the same (1∶1) at all levels of US28 signaling tested. For example at the lowest level of US28 expression (1000pg/ml DOX), CCL5/RANTES had no effect on PLC-β signaling as the amount of inositol phosphate conversion was 1.8% versus 1.6% when comparing plus or minus CCL5/RANTES. Thus, taken together, these studies indicate that US28 is an extremely potent agonist-independent molecule likely to have significant effects on cellular signaling regardless of its expression level.

Analysis of US28 signaling and biological activity in primary endothelial cells has been hampered by the fact that model cell systems such as HUVECs are very difficult to efficiently infect and because many of the HCMV strains for which recombination technologies are available do not infect this cell type. Infection and replication within endothelial cells is very relevant *in vivo* and since there is a unique repertoire of viral genes needed for replication in endothelial cells, we wished to investigate US28 signaling and its affect on viral replication in HUVECs. We used our newly constructed TB40/E-based recombinants and found that US28 is in fact expressed in this cell type, generates an agonist-independent signal, but is not required for immediate early or early protein expression. Moreover, we found that US28 is not required for efficient virus production in endothelial cells as the wild-type and ΔUS28 viruses replicate to similar titers. Thus, similar to previous observations in fibroblasts, it is now clear that US28 is not absolutely required for replication in either fibroblasts or endothelial cells.

HCMV encodes four different GPCRs, US27, US28, UL33, and UL78 presenting the possibility for redundancy within this gene family that obscures essential functions for the individual GPCRs under different conditions. A recombinant virus deleted for all four GPCRs (ΔALL) exhibits a defect in replication in both fibroblasts and endothelial cells. While this defect is likely due, at least in part, to the deletion of US27 in the case of fibroblasts and HUVECs [44] as well as the deletion of UL78 in the case of endothelial cells [60], this TB40/E-ΔALL virus confirms that the viral GPCRs provide functions important for replication *in vitro*. Moreover, this recombinant also provides an interesting backbone in which to investigate questions regarding redundancy and receptor co-operation by constructing single put-backs on an otherwise GPCR null background. For example, this derivative will serve as an important platform to determine if the individual GPCRs such as US28 can rescue cell-type specific growth and also determine if US28 is a competent signaling molecule when expressed in isolation during HCMV infection.

In summary, we report the first comprehensive molecular analyses of US28 signaling during infection of a variety of cell types important for HCMV pathogenesis. The data indicate that US28 is ubiquitously expressed in all cell types tested and exhibits potent agonist-independent signaling through PLC-β. Moreover, US28 exhibits cell-specific signaling activities and has robust effects on signaling via endogenous GPCRs, making for a very diverse potential number of signaling pathways affected. These studies provide the platform to further examine how these signals proceed downstream and how they affect other aspects of signaling in infected cells. Finally, we describe a number of new viral mutants that can be used to further dissect US28 signaling and determine the impact that this viral encoded GPCR has on cellular regulation and pathogenesis.

## Supporting Information

Figure S1 **CCL5/RANTES does not affect US28 stimulated PLC-β activity.** HASMCs were infected with the indicated viruses at a MOI of 3 and the ability of FIX-US28Flag and each of the US28 mutant viruses to activate PLC-β was assessed at 48 hpi. PLC-β activation was measured by labeling cells 24 hpi with 1 µCi/ml 3H-myoinositol. Inositol phosphates were isolated at 48 hpi and PLC-β activity is represented as the percent conversion of input myoinositol into inositol phosphates.
(TIF)

Figure S2 **HCMV infection promotes increased intracellular calcium that can be driven higher in the presence of US28 and CCL5/RANTES.** HASMCs were infected with FIX-US28Flag or FIX-ΔUS28 viruses at a MOI of 3. At 48 hpi, cells were labeled with Fluo-4 AM, stimulated with 10 nM CCL5/RANTES and total intracellular calcium was measured using a FlexStation II fluorometer. The data is plotted as raw fluorescence to demonstrate actual intracellular calcium levels at the time agonist was added. The traces are representative of at least three independent experiments performed in duplicate.
(TIF)

Figure S3 **Infectivity rates of TB40/E recombinant viruses in HFFs and HUVECs.** In parallel to experiments shown in Figure 8A, HS68 HFFs infected with MOIs of 0.2, 1, and 5 (left panel) or HUVECs infected with MOIs of 0.6, 3, and 15 (right panel) were analyzed by flow cytometry for mCherry expression to assess infection rates with the different viruses.
(TIF)

Table S1 **Primers used for generating TB40/E recombinants.** DNA sequences of primers used for PCR amplification of *galK* containing recombination fragments and DNA sequences of double stranded oligos used for subsequent deletion of *galK* sequences are depicted.
(DOCX)
