# Supplementary figures and images for: US28 Is a Potent Activator of Phospholipase C during HCMV Infection of Clinically Relevant Target Cells
Source: PLoS One. 2012 Nov 29;7(11):e50524. doi: 10.1371/journal.pone.0050524 (PMC3510093; doi:10.1371/journal.pone.0050524)

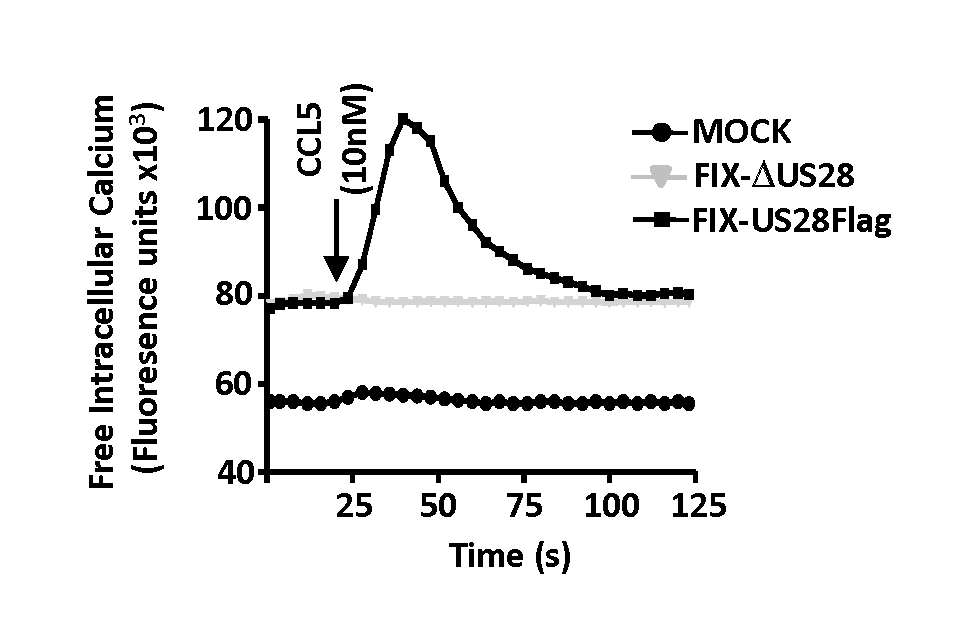

Supplement: Figure S2 — HCMV infection promotes increased intracellular calcium that can be driven higher in the presence of US28 and CCL5/RANTES. HASMCs were infected with FIX-US28Flag or FIX-ΔUS28 viruses at a MOI of 3. At 48 hpi, cells were labeled with Fluo-4 AM, stimulated with 10 nM CCL5/RANTES and total intracellular calcium was measured using a FlexStation II fluorometer. The data is plotted as raw fluorescence to demonstrate actual intracellular calcium levels at the time agonist was added. The traces are representative of at least three independent experiments performed in duplicate. (TIF) [file pone.0050524.s002.tif]
