# Supplementary material for: US28 Is a Potent Activator of Phospholipase C during HCMV Infection of Clinically Relevant Target Cells
Source: PLoS One. 2012 Nov 29;7(11):e50524. doi: 10.1371/journal.pone.0050524 (PMC3510093; doi:10.1371/journal.pone.0050524)
Supplement: Table S1 — Primers used for generating TB40/E recombinants. DNA sequences of primers used for PCR amplification of galK containing recombination fragments and DNA sequences of double stranded oligos used for subsequent deletion of galK sequences are depicted. (DOCX) [file pone.0050524.s004.docx]

| **Supplemental Table 1. Primers used for generating TB40/E recombinants.** | | |
| --- | --- | --- |
| **Recombinant Virus (TB40/E)** | **Primer Direction** | **Primer Sequence (5'**🡺**3')** |
| US28FLAG-kan- frt insertion | FOR | TCTTCCGACACGCTGTCCGACGAGGTGTGTCGCGTCTCACAAATTATACCGttagattataaagatgatgatgataaa**^§^** |
|  | REV | AGAGGGGCGGACACGGGGTTTGTATGAAAAGGCCGAGGTAGCGCTTTTTTAggccgcgggaattcgaagtt**^§^** |
| US28Δ-*galK* insertion | FOR | GGTGCGTGGACCAGACGGCGTCCATGCACCGAGGGCAGAACTGGTGCTATCcctgttgacaattaatcatcggca**^#^** |
|  | REV | AGAGGGGCGGACACGGGGTTTGTATGAAAAGGCCGAGGTAGCGCTTTTTTAtcagcactgtcctgctcctt**^#^** |
| US28Δ | ds oligo | CAGACGGCGTCCATGCACCGAGGGCAGAACTGGTGCTATCTAAAAAAGCGCTACCTCGGCCTTTTCATACAAACCCCGTG |
| UL33Δ-*galK* insertion | FOR | TTCCGCCCAGACCCGCAACAACACTCCTCCGCACATCAATGACACTTGCAACcctgttgacaattaatcatcggca**^#^** |
|  | REV | GGGGAAATGGCGACGGGTTCTGGTGCTTTCTGAATAAAGTAACAGGAAAGCtcagcactgtcctgctcctt**^#^** |
| UL33Δ | ds oligo | CCGCAACAACACTCCTCCGCACATCAATGACACTTGCAACGCTTTCCTGTTACTTTATTCAGAAAGCACCAGAACCCGTC |
| UL78Δ-*galK* insertion | FOR | GTCCCCGGAGAGGGTATATTCGTTCGGCGAGAGCGGGCGGCGGTGGTGGGTcctgttgacaattaatcatcggca**^#^** |
|  | REV | TAACGTGATTTATCTGCCACTTTTCTCCCCGCTGCCGTACAGCGCCGCCGCtcagcactgtcctgctcctt**^#^** |
| UL78Δ | ds oligo | GGGTATATTCGTTCGGCGAGAGCGGGCGGCGGTGGTGGGTGCGGCGGCGCTGTACGGCAGCGGGGAGAAAAGTGGCAGAT |
| US27Δ:US28Δ-*galK* insertion | FOR | GTGTAATGCTTTTTACAGGACCGTTCAACAGGTGATACTACCTGCAAGGTAcctgttgacaattaatcatcggca**^#^** |
|  | REV | AGAGGGGCGGACACGGGGTTTGTATGAAAAGGCCGAGGTAGCGCTTTTTTAtcagcactgtcctgctcctt**^#^** |
| US27Δ:US28Δ | ds oligo | TTTACAGGACCGTTCAACAGGTGATACTACCTGCAAGGTATAAAAAAGCGCTACCTCGGCCTTTTCATACAAACCCCGTG |
| **^§^**Lowercase sequences are complementary to the pGTE-3xFLAG-Kan-frt template. | | |
| **^#^**Lowercase sequences are complementary to the *galK* template plasmid. | | |
